# Horses cross-modally recognize women and men

**DOI:** 10.1038/s41598-023-30830-6

**Published:** 2023-03-08

**Authors:** Chloé Gouyet, Monamie Ringhofer, Shinya Yamamoto, Plotine Jardat, Céline Parias, Fabrice Reigner, Ludovic Calandreau, Léa Lansade

**Affiliations:** 1 CNRS, IFCE, INRAE, Université de Tours, PRC, 37380 Nouzilly, France; 2 Department of Animal Science, Teikyo University of Science, Yamanashi, Japan; 3 Institute for Advanced Study, Kyoto University, Kyoto, Japan; 4 Wildlife Research Center, Kyoto University, Kyoto, Japan; 5 UEPAO, INRAE, 37380 Nouzilly, France

**Keywords:** Zoology, Animal behaviour

## Abstract

Several studies have shown that horses have the ability to cross-modally recognize humans by associating their voice with their physical appearance. However, it remains unclear whether horses are able to differentiate humans according to different criteria, such as the fact that they are women or men. Horses might recognize some human characteristics, such as sex, and use these characteristics to classify them into different categories. The aim of this study was to explore whether domesticated horses are able to cross-modally recognize women and men according to visual and auditory cues, using a preferential looking paradigm. We simultaneously presented two videos of women and men’s faces, while playing a recording of a human voice belonging to one of these two categories through a loudspeaker. The results showed that the horses looked significantly more towards the congruent video than towards the incongruent video, suggesting that they are able to associate women’s voices with women’s faces and men’s voices with men’s faces. Further investigation is necessary to determine the mechanism underlying this recognition, as it might be interesting to determine which characteristics horses use to categorize humans. These results suggest a novel perspective that could allow us to better understand how horses perceive humans.

## Introduction

Horses (*Equus caballus*) have advanced social cognition abilities, especially in relation to their interaction with humans [e.g.,^1–8^]. Individual recognition is important in social interactions, and horses are able to discriminate between humans based on visual or vocal cues, similar to many other domesticated species, such as cats, cows, dogs, horses, pigs and sheep^9^. Previous studies have demonstrated that horses spontaneously recognize their handler, last seen six months prior, from a picture^10^. It seems that horses do not solely rely on an easy cue, such as hairstyle, for recognition, suggesting that face recognition is a holistic process. Indeed, horses are also able to link faces from photographs to people in real life, indicating that horses do not process photographs of human faces as simple abstract shapes^11^. In a study investigating the cross-modal categorization of human emotions, horses matched visual and vocal cues for the same emotion (joy or anger)^12^. Horses also use multimodal cues to recognize familiar humans. In one study, horses were visited by a familiar person who then passed out of the horse’s field of vision; the horses were then more surprised to hear a playblack of another human’s voice (of the same sex) than that of the familiar visitor. This suggests that horses are able to make the connection between the person they just saw and the voice that they heard^13^. In another study, horses were demonstrated to be capable of cross-modal recognition of familiar individuals. When placed in front of two people (of the same sex), they looked preferentially towards the person corresponding to the voice being broadcast^14^. However, horses might use different human characteristics to rely on for recognition. For instance, horses might categorize humans according to their age, sex or size. In a recent study, horses associated children’s voices with children’s faces and adults’ voices with adults’ faces^15^. This present study now focuses on recognition of men and women.

In humans, there are differences between women and men in both physical appearance and voice^16^, although there are some exceptions to this general rule. These differences emerge by sexual maturity, during puberty and adolescence^17^; thus, women and men are sexually dimorphic^18^. A study conducted by O’Toole and collaborators in 1998^19^ reported that the faces of women and men in photographs were correctly categorized 95% of the time in their database. In addition, the voices of adult women and men differ. In average the fundamental frequency is 120 Hz for women’s voices and 200 Hz for men’s voices^20^. Women retain higher frequencies than men^21^. This disparity is due to sexual dimorphism in the vocal tract anatomy of adults, as men have a larger larynx^22^. After adolescence, men have also more resonant voices due to their longer vocal tract^23^. As well as individual behaviour, the vocal expression of masculinity or femininity can be controlled even before puberty, before the anatomical difference in the vocal apparatus emerges^24^.

Different species seem to be able to use these characteristics to discriminate between and modify their behaviour towards women and men. African elephants (*Loxodonta africana*) categorize humans on the basis of visual, auditory and olfactory cues^25^. For example, they categorize humans according to the level of threat they represent: as they are hunted only by men, elephants discriminate humans based on sex because this characteristic can dramatically affect predation risk^26^. Thus, since certain humans are predators for these elephants, it is vital that they develop the ability to discriminate between categories of humans to recognize the most dangerous ones. Dogs also seem to be able to discriminate between women and men. They discriminate between two individuals more easily if those two persons are of different sexes^27^. Moreover, several studies have reported that dogs differ in their interactions with women and men: they bark less at women and look more towards men^28^, display more stress-related behaviours when interacting with men^29^, and show more relaxed behaviours and lower cortisol levels when petted by women than by men^30^. Moreover, male dogs were less likely to approach and make body contact with an unfamiliar man^31^ and urinated more when walked by unfamiliar women than when walked by unfamiliar men^32^. In addition, dogs cross-modally recognize people according to sex: when hearing a voice, dogs expect to see an appropriate sex-matched visual cue^33^. To date, it remains unknown whether horses that are regularly in contact with humans are able to cross-modally recognize women and men. Thus, we sought to evaluate this ability in the current study.

This study aimed to investigate whether horses are able to associate a woman’s voice with a woman’s face and a man’s voice with a man’s face, using a preferential looking paradigm. We based our protocol on one already used successfully with horses to explore cross-modal recognition of human emotions^12^ and cross-modal recognition of children and adults^15^. Two mute videos of people talking (one of a woman’s face, one of a man’s face) were simultaneously presented to the horse while a woman’s voice or a man’s voice was broadcast, this was repeated in 6 trials with different stimuli. Our hypothesis was that horses would look preferentially at one of the videos depending on the vocal stimulus. We analysed the gaze duration, the latency to the first look and the number of looks towards the videos. We did not predict the gaze direction as, according to the literature, gaze can either be directed towards the congruent stimulus^14^ or the incongruent stimulus^12,15^. To determine if hearing women’s voices or men’s voices induced different emotional reactions in the horses, we compared their variation in heart rate and the behavioural signs of emotion (e.g., defecating, shaking their head, pawing at the ground, rearing and vocalizing) while hearing the two types of voices. Indeed previous studies, using a similar protocol, noticed that horses’ heart rate increased more while hearing children’s voices than adults’ voices^15^ and anger vocalizations than joy vocalizations^12^. As animals can sometimes display different kind of reactions towards women and men^26,28–32^, the type of stimuli might affect differentially horses’ reactions.

## Materials and methods

### Subjects

This study included forty Welsh mares aged 4–13 years old (mean age ± SD = 8.94 ± 2.45). The mares were born and bred at the experimental unit of INRAE (PAO, INRAE, Nouzilly, France, Animal Physiology Experimental Facility, https://doi.org/10.15454/1.5573896321728955E12). Those horses were only used for research purposes. They were kept in herds in a pasture in spring and summer or in large stables with straw bedding and daily access to an outside paddock during autumn and winter. The experiment took place in winter. Hay and water were available ad libitum. These horses were fully habituated to humans. All the horses were introduced to the experimental setup during familiarization sessions. Nine horses were excluded from the experiment since they did not reach the fixed criterion of the familiarization sessions (see the Familiarization with the Experimental Setup section). Therefore, thirty-one horses were included in the final testing.

### Experimental design

**Figure 1:**
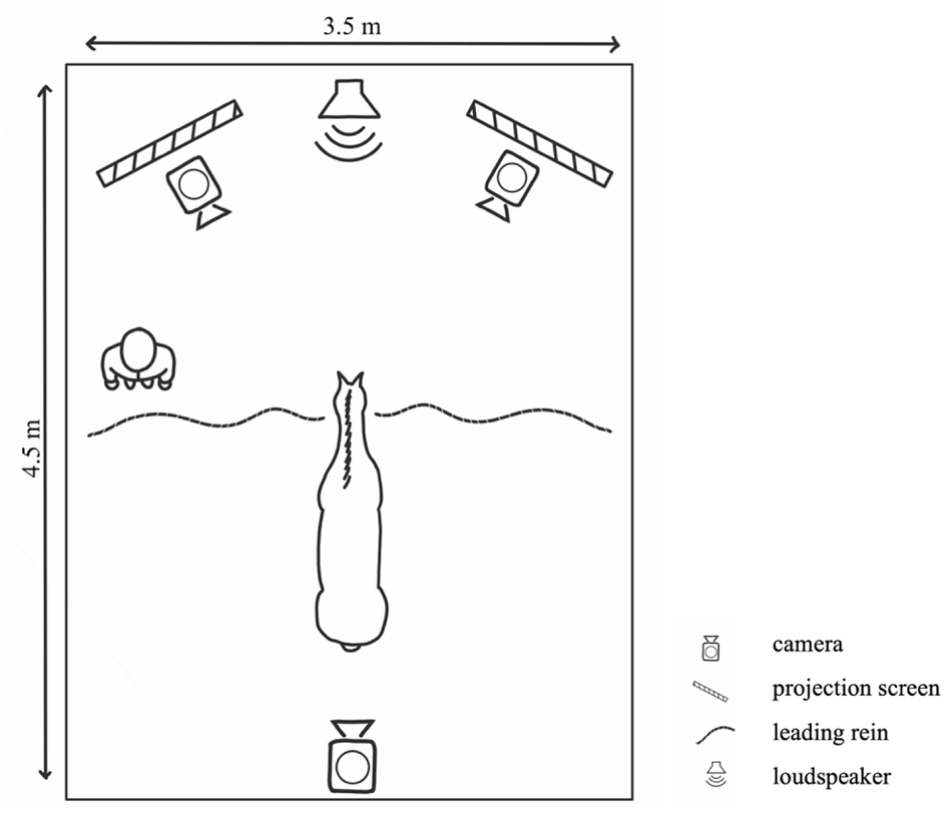
Schematic representation of the experimental design.

Horses were tested individually in a stall (3.5 × 4.5 m, Fig. 1). They were placed in front of two white projection screens (1 × 2 m) located in the right and left corners of the stall. A loudspeaker was located between the projection screens, in front of the horse.

During testing, the horses were attached with two leading reins, one on each side of the head. For safety reasons, an assistant stayed next to the horse during testing. The assistant kept his back to the projection screens and looked at the ground, but was ready to intervene in case the horse reacted in an extreme or dangerous manner. The side of the horse (right or left) that he stood was counterbalanced between horses. The assistant was blinded to which videos were played on the two projection screens so as not to influence the horse via unintentional cues.

One camera was placed under each projection screen to record the horse’s behaviours. A third camera was placed up high behind the horse to provide an overview of testing; this camera allowed the experimenter to watch and supervise the test sessions from outside the stall. Additionally, the horses were equipped with a heart monitoring system (Polar Equine RS800CX Science, Polar Oy, Finland) to measure their heart rate.

### Familiarization with the experimental setup

Before the experiment, the horses were familiarized with the experimental setup. The familiarization sessions took place in the mornings. During the familiarization sessions, videos of landscapes were presented on the two projection screens, while the loudspeaker played recordings of birdsong. Horses underwent at least 5 min of familiarization. Then, the assistant checked the heart rate of the horse. The familiarization session stopped when the horse reached the determined criterion: the horse exhibited a heart rate lower than 80 bpm for at least one minute and stood in front of the projection screen for one minute without requiring any assistant intervention. The familiarization session lasted for a maximum of 10 min. If the horses reached the criterion in the morning, they were tested in the afternoon. If the horses did not reach the criterion, they were familiarized again the next morning. If the horses did not reach the criterion during the two familiarization sessions, they were excluded from the experiment. In total, nine horses were excluded. With more familiarization sessions, these horses would have probably reached the criterion. However, we decided to limit the number of sessions to homogenize the testing conditions between the horses; and also for ethical reasons (if the horses were not comfortable in front of the screen, we preferred to not insist).

### Test procedure

The test sessions took place in the afternoons. The test sessions consisted of 6 trials. During each trial, two mute videos were played simultaneously, one on each projection screen, while a voice was played from the loudspeaker placed between the projection screens. Specifically, the two mute videos consisted of one video woman’s video and one man’s video, while the voice consisted of a repeated sentence said by a woman or man. The stimuli (videos and voices) presented to the horses were different in the 6 trials. In addition, the voice played was not the original voice of one of the two people in the two videos to avoid any bias due to the horses potentially matching the mouth movements with the voice they were listening to.

**Figure 2:**
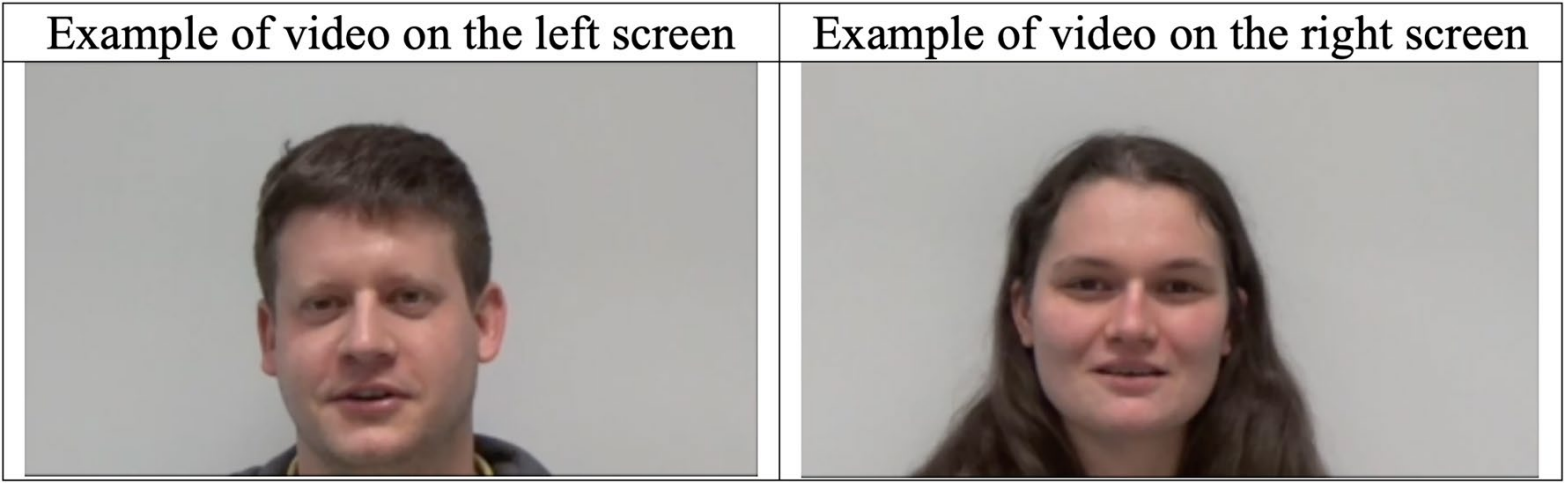
Screenshots of 2 videos presented simultaneously during 1 trial. The videos change at each trial (thus each horse saw 6 different women and 6 different men).

All the stimuli were recorded under the same conditions in the same room at INRAE. The videos were recordings of the faces (Fig. 2) of 12 people, we recorded 6 men and 6 women aged 23–34 years old. During the recording of the video stimuli, the people remained motionless and faced the camera, placed 2 m away, with their backs against a white wall. People had to say a neutral sentence. The sentence was simple: “I like pistachio ice cream; I like chocolate ice cream” said in french (“J’aime la glace à la pistache; j’aime la glace au chocolat”). The audio part was recorded at the same time. 6 recorded voices were kept (3 women’s voices and 3 men’s voices) to have one voice for each trial.

For each trial, the couple of videos and the voice were repeated 4 times for a total duration of 16 s. Between each trial was a 5-s break in which the screens were black and no sound played. Therefore, the test had a total duration of 121 s. Horses underwent 6 trials in total: 3 trials with a woman’s voice and 3 trials with a man’s voice. The order and sides of the women and men’s videos were counterbalanced between horses. Additionally, the order of the voice (man’s or woman’s voice) and the side of the congruent video were semirandomly distributed between trials, such that videos that were congruent with the voice were played an equal number of times on the right side and on the left side for each horse.

The videos were validated by 20 persons who categorized women and men with 100% accuracy. The vocal parameters of the recordings were analysed using PRAAT v.5.0.3 (http://www.fon.hum.uva.nl/praat/). Fundamental frequencies (F0) were calculated using the PRAAT algorithm *Pitch*. The mean F0 for the men’s voices was 140 Hz (+ SE = 24), while the mean F0 for the women’s voices was 215 Hz (+ SE = 19). The voice was played from the loudspeaker at a level of 60-70 dB according to the location of the horse.

### Data collection

#### Gaze responses

The horses’ gaze direction towards the two different projection screens during playback events was analysed. Horses were considered to be looking at a video if they turned their head towards the projection screen. One camera was placed under each projection screen. Horses were considered to look at the video when their faces were directed towards the camera such that the observer could see their opposite eye. Thus, in this preferential looking paradigm, the gazes towards the congruent and incongruent videos for each trial were analysed.

To avoid bias due to lateralization, horses were excluded from the analysis if they did not meet a criterion. Indeed, some horses looked almost exclusively at one projection screen (either placed on their left or on their right, independent of the congruence of the video presented). To avoid this bias, the following criterion was established: during the test sessions, if a horse directed less than 10% of the total gaze duration to one side, they were excluded from the analysis. Therefore, four additional horses were excluded from the analysis.

Next, we analysed three variables to investigate the gaze responses and calculate the preference indexes: (INC − CON)/(INC + CON).

1. Gaze duration: the abbreviation INC corresponds to the total gaze duration directed towards the incongruent videos, and the abbreviation CON corresponds to the total gaze duration directed towards the congruent videos.

2. Latency to the first look: the abbreviation INC corresponds to the duration from the beginning of the trial to the first look directed towards the incongruent video, and CON corresponds to the duration from the beginning of the trial to the first look directed towards the congruent video.

3. Number of looks: the abbreviation INC corresponds to the number of looks directed towards the incongruent videos and the abbreviation CON corresponds to the number of looks directed towards the congruent videos.

For each individual, the mean of the indexes obtained for each trial was calculated to obtain a mean preference index. The mean preference indexes were between − 1 and 1. If the mean preference index equaled 0, the horse looked equally at the congruent and incongruent videos.

All the videos were analysed by the same observer. The observer was blinded to the voice played and analysed the videos without sound. A second observer analysed 20% of the videos. The interobserver reliability was calculated with the interclass correlation coefficient (ICC)^34^. The ICC for the gaze duration had a lower bound of 0.91 and an estimate of 0.94, which indicated excellent interobserver reliability. The ICC for the latency to the first look had a lower bound of 0.99 and an estimate of 0.99, which is also considered excellent interobserver reliability. The ICC for the number of looks had a lower bound of 0.85 and an estimate of 0.90, which is considered good interobserver reliability.

#### Additional behaviours

In addition, the following behaviours were also recorded to assess the emotional response of horses: defecating, shaking their head, pawing the ground and rearing. All horse vocalizations were also recorded. However, too few horses engaged in these additional behaviours to permit statistical analysis (defecating: *N* = 3, shaking their head: *N* = 2, pawing the ground: *N* = 1, rearing: *N* = 0, vocalizing: *N* = 3).

#### Physiological responses

For each trial, the differences in the mean heart rate between the last 5 s and the first 5 s were calculated. Calculating this difference allowed us to determine whether the heart rate of horses increased or decreased while hearing the different voices. Heart rate data for two individuals were missing due to technical issues with the monitoring system.

### Statistical analysis

The statistical analysis was carried out using RStudio 3.3.6 statistics software. A comparison was made between the mean preference indexes and the theoretical value corresponding to a random choice obtained by chance (*mu* = 0). The normality of the distribution of variables was assessed graphically using the function *qqPlo*t in the package *car*. Data were analysed with a parametric test: a bilateral one sample Student’s *t* test using the function *t.test*. The heart rate responses to the voices (man’s or woman’s voice) were analysed with a paired Student’s *t* test. The significance threshold throughout this study was set at *P* < 0.05.

### Ethical note

We had the permission to use animals and humans for experimental purposes as the study was approved by the Val de Loire Ethical Committee (Authorization number: CE19-2021-3011-1, CEEA VdL, Nouzilly, France). Animal care and experimental treatments complied with the French and European guidelines for the housing and care of animals used for scientific purposes (European Union Directive 2010/63/EU) and were performed under authorization and supervision of an official veterinary of the Département d’Indre et Loire (France). The subjects were not food deprived during the experiment. They lived in social groups and had daily access to an outside paddock. The experiment collected only noninvasive data. All methods were performed in accordance with relevant guidelines and regulations. Written informed consent was obtained from all the volunteers for their study participation and written informed consent for publication of images in an open-access was obtained for two of the participants.

## Results

**Figure 3:**
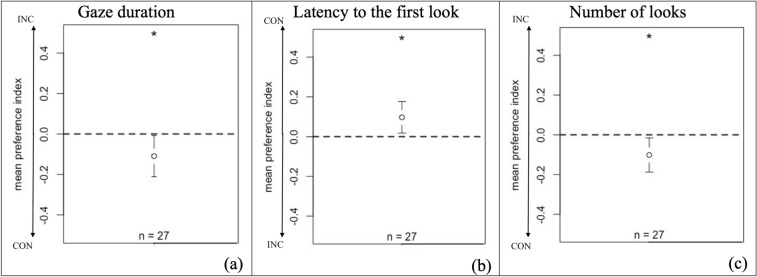
Mean preference index per individual: (INC − CON)/(INC + CON) for each trial. The plot shows the group mean and the confidence interval. Significance was assessed by a Student’s *t* test: * = *P* < 0.05. The arrows show the preference of the horses toward the congruent or incongruent video. The dotted line corresponds to random choices (indicating a lack of preference).

The mean preference index per individual was (INC − CON)/(INC + CON) for each trial. On the plot: for the gaze duration (Fig. 3a) and the number of looks (Fig. 3c), the closer the mean preference index is to  − 1, the longer horses looked at the congruent video, thus negative values indicate a preference for the congruent stimulus; for the latency of the first look (Fig. 3b), the closer the mean preference index is to 1, the faster horses looked at the congruent video, thus positive values indicate a preference for the congruent stimulus.

The mean preference indexes for the gaze duration were significantly different from 0 (Student’s test: *t* =  − 2.212, *P* = 0.036; Fig. 3a). Horses directed their gaze more towards the congruent video than the incongruent video. The mean indexes for the latency to the first look were also significantly different from 0 (Student’s test: *t* = -2.522, *P* = 0.018; Fig. 3b). Horses directed their first looks more towards the congruent video than the incongruent video. The mean indexes for the number of looks were significantly different from 0 (Student’s test: *t* =  − 2.4254, *P* = 0.023; Fig. 3c). Horses looked more frequently towards the congruent video than the incongruent video.

**Figure 4:**
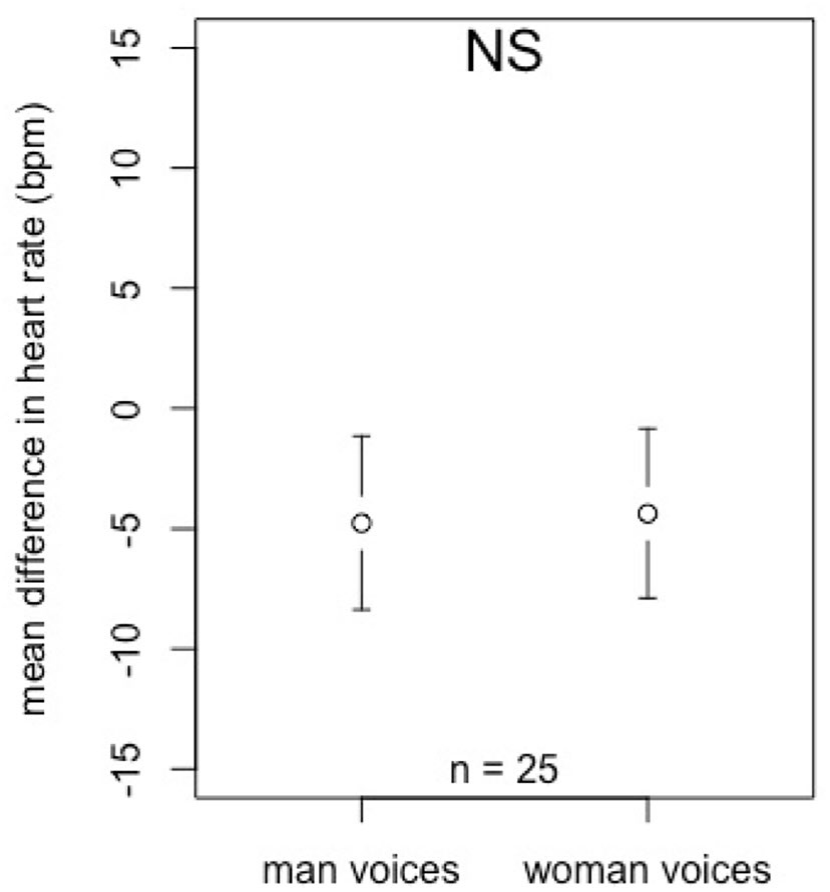
Effect of the voice (man’s or woman’s voice) on the heart rate of horses: mean individual differences between the mean heart rate of the last 5 s and the first 5 s of each trial. The plot shows the group means and the confidence interval. Significance was assessed by a Student’s *t* test.

The change in the heart rate of horses did not significantly (NS) differ between women and men’s voices (Student’s test: *t* =  − 0.079, *P* = 0.869; Fig. 4).

## Discussion

Horses gazed longer towards the congruent video: while hearing a woman’s voice, horses gazed more towards the woman’s video, and while hearing a man’s voice, they gazed more towards the man’s video. Moreover, horses were more likely to look first towards the congruent video, and they looked at the congruent video more frequently. Therefore, horses seem to associate women’s voices with women’s faces and men’s voices with men’s faces, suggesting that they utilize cross-modal cues to recognize humans. The heart rate of horses did not differ between the two types of voices.

In this experiment, horses looked preferentially at the congruent stimuli. In similar preferential looking paradigms, some studies have indicated that subjects looked more towards the congruent stimuli, while others found the opposite. Our results are in line with a study by Proops and McComb done in 2012^14^, which investigated the cross-modal categorization of familiar humans; in this study, horses spent more time looking at congruent stimuli. Conversely, in the study investigating the cross-modal categorization of children and adults^15^ and the one investigating cross-modal categorization of human emotions^12^, horses spent more time looking at the video that was incongruent with the sound. A possible explanation for this discrepancy could be related to the nature of the stimuli, especially to the emotion that they induced. In our experiment, the two types of stimuli did not seem to induce differently any specific emotion, based on the heart rate response. In contrast, in Trösch and collaborators’ study^12^, the heart rate of horses increased when they heard angry vocalizations, suggesting that these stimuli may have worried or surprised horses. In Jardat and collaborators’ study^15^, the heart rate of horses increased when they heard children’s voices, as the horses used in this study had never seen a child in their whole life, they might have been more stressed or surprised by voices with unfamiliar characteristics. This explanation is supported by another study in dogs^33^ that investigated their ability to cross-modally categorize humans according to sex. On the one hand, dogs living with both a woman and a man (and thus familiar with both sexes) looked more at the congruent person; on the other hand, when living with only one person, either a woman or a man, they looked more at the incongruent person. Thus, subjects might preferentially look at the congruent video when the stimulus does not induce any specific emotion (e.g., when stimuli are familiar) and look at the incongruent video when one stimulus induces worry or surprise. However, this potential explanation needs further investigation.

Furthermore, in our study, we tried to avoid inducing biases. First, we used various faces and voices; thus, the results are not individual dependent. Second, this study was a playback experiment, and the voices played while projecting the videos were not the original voices of any of the two persons in the videos presented to avoid the possibility that horses might match the mouth movements to the voices. However, additional investigations are needed to better understand which cues horses use to categorize woman and man stimuli. Women and men’s faces generally differ on numerous characteristics, such as shape or skin texture^35^, and their voices differ not only in frequency but also phonetically^36^. Horses could potentially form a holistic representation that takes all of these characteristics into account to categorize women and men. In a previous experiment, we showed that horses recognize a familiar human face in photographs despite modifications such as changing the colour to black and white, changing the angle, hiding the eyes or using different hairstyles^11^, which suggests that horses employ a holistic process for facial recognition. Nevertheless, the categorization of individuals into women and men may be based on a few specific cues and this study has limitations. We presented 6 women’s faces and 6 men’s faces, twelve people to have different person in each trial. However, the participants were all Caucasian in the same age range, and, as an example, all women had long hair, unlike men. Horses could simply associate high vocal frequencies with longer hair to distinguish between the women and men presented in this experiment. It would be interesting to generalize the result with other people.

Nevertheless, our study has some limitations. We chose to calculate indexes (INC − CON)/(INC + CON) to take into account the variability of the total gazing time between horses. Then, we compared these indexes to a theoretical value (zero), as it had been done in previous studies using the same protocol^12,15^. This approach prevents us from addressing potential confounding variables such as the side of the person who stood next to the horses and the order of presentation of the stimuli. These variables were counterbalanced beforehand between horses to avoid any bias, however to not include these factors in the statistical analyses is a limitation of our approach and we must remain careful about the interpretation of the results.

## Conclusion

Horses are able to cross-modally recognize women and men: they associate women’s faces with women’s voices and men’s faces with men’s voices. Future investigations are needed to determine which characteristics horses use to categorize humans and whether they use simple cues or a more holistic process. These findings provide a novel perspective that may allow us to better understand how horses perceive humans. Indeed, they reveal that horses can effectively cross-modally categorize women and men. Specifically, that suggests that horses may generalize their experiences with one person to other people in the same category (i.e. women or men). Horses remember previous interactions with humans and act consequently in future interactions^37^. In this study, the use of a reward during a learning task induced positive reactions toward the human, and increased contact and interest toward that human several months later. It would be interesting to explore how horses use this information to adjust their behavior towards different individuals depending on their category membership. This knowledge could enhance horse training techniques and enhance the safety and welfare of both horses and humans.

## Data Availability

The datasets generated and analysed during the current study are available in the INRAE data repository: https://entrepot.recherche.data.gouv.fr/dataset.xhtml;jsessionid=a1163ebabefeaecff796c2b3e6b4?persistentId=doi%3A10.57745%2FND0SNH&version=DRAFT.
